# Normalizing RNA-Sequencing Data by Modeling Hidden Covariates with Prior Knowledge

**DOI:** 10.1371/journal.pone.0068141

**Published:** 2013-07-18

**Authors:** Sara Mostafavi, Alexis Battle, Xiaowei Zhu, Alexander E. Urban, Douglas Levinson, Stephen B. Montgomery, Daphne Koller

**Affiliations:** 1 Department of Computer Science, Stanford University, Stanford, California, United States of America; 2 Department of Psychiatry & Behavioral Science, Stanford University, Stanford, California, United States of America; 3 Department of Pathology, Stanford University, Stanford, California, United States of America; University of Pittsburgh, United States of America

## Abstract

Transcriptomic assays that measure expression levels are widely used to study the manifestation of environmental or genetic variations in cellular processes. RNA-sequencing in particular has the potential to considerably improve such understanding because of its capacity to assay the entire transcriptome, including novel transcriptional events. However, as with earlier expression assays, analysis of RNA-sequencing data requires carefully accounting for factors that may introduce systematic, confounding variability in the expression measurements, resulting in spurious correlations. Here, we consider the problem of modeling and removing the effects of known and hidden confounding factors from RNA-sequencing data. We describe a unified residual framework that encapsulates existing approaches, and using this framework, present a novel method, HCP (Hidden Covariates with Prior). HCP uses a more informed assumption about the confounding factors, and performs as well or better than existing approaches while having a much lower computational cost. Our experiments demonstrate that accounting for known and hidden factors with appropriate models improves the quality of RNA-sequencing data in two very different tasks: detecting genetic variations that are associated with nearby expression variations (cis-eQTLs), and constructing accurate co-expression networks.

## Introduction

The transcriptional landscape of a cell is complex and with the help of RNA-sequencing technology (RNA-seq) we are now starting to characterize it. In particular, RNA-seq technology can assay the entire transcriptome, including known isoforms of each gene and even novel transcripts. Combined with genotyping assays, we can more accurately elucidate the genetics of the transcriptome by identifying diverse transcriptional events that are associated with genetic variants. (Such associated variants are referred to as eQTLs). In this way we can begin to precisely identify the molecular mechanisms that underlie functional genetic variations, including implicated disease loci from genome-wide association studies, and prioritize implicated hits for follow-up experiments [1].

However, quantifying expression levels from RNA-seq data is not a trivial task: the output of an RNA-seq assay is millions of short RNA sequences, which then must be mapped to the genome and aggregated to quantify the expression level of each transcript. The accuracy of the resulting expression level estimates depends not only on the mapping and quantification algorithms, but also on carefully correcting for several technological biases that result in unwanted variability in the number of reads for each transcript. For example, technical factors involved in sample collection, library preparation, and sequencing may manifest as variation that depends on the batch, date, or sequencing lane of a sample. Other well-known examples of unwanted technical variability include sequencing biases related to GC content [2], and variation due to limited sequencing depths (SD) [3].

In addition to technical artifacts from measured, known, covariates such as sequencing depth, it may be useful to account for “hidden” covariates (or factors) that can introduce variability into the transcriptome. Hidden factors may comprise technical artifacts from unmeasured differences in sample preparation, while others arise from factors not specific to RNA-seq technology [4]. For example, unwanted, or unknown biological variations in the subject pool, such as in BMI, can have broad effects on gene expression [5], resulting in biological variability that confounds identification of the effectors of interest, such as disease causing genes or eQTLs [6].

Such known and hidden systematic biases result in spurious correlations between subjects or certain transcripts, and depending on the type of analysis that is performed, lead to increased false positives or false negatives. For instance, it is well known that spurious correlations among subjects decreases the power to detect eQTLs [7], [8], and can also result in false associations [9]. Similarly, co-expression networks constructed from uncorrected data will likely include many false positive edges due to broad trends in the data [10]. Normalizing RNA-seq measurements to account for both known and hidden biases is a central task in the analysis of such data. Indeed, several methods have been proposed for normalizing microarray data by attempting to account for both known and hidden covariates, and these methods have been shown to dramatically improve various down-stream analyses [6]–[8], [10], [11] – resulting, for example, in a two- to three-fold increase in the number of statistically significant associations between SNPs and nearby transcripts (known as cis-eQTL) [7], [12], as well as significantly enhanced reproducibility of results among different datasets [12]. However, to date, such methods have not been applied to RNA-seq data.

In this paper, we first describe a unified *residual* normalization framework encompassing most existing methods that attempt to account for both known and hidden covariates. In particular, existing normalization methods essentially combine a linear dimensionality reduction step to estimate the hidden covariates through matrix factorization, and a regression of the expression data onto covariates constructed from both known and hidden covariates. The way in which each of these two steps are optimized, and combined, differentiates the various approaches. This unified framework allows us to better compare existing approaches, and identify components whose variation is linked to specific underlying biological assumptions. For example, all methods derived from this framework assume that hidden covariates, like principal components, represent broad patterns in expression that affect many genes in the same way – an assumption likely to fail when the phenotype of interest is also expected to have a strong effect on expression patterns (e.g., [13]).

To go beyond the limiting assumption of existing methods, we formulate a new model of hidden co-variation, and develop the HCP (Hidden Covariates with Prior) method to renormalize the two largest RNA-seq data sets constructed to date [2], [14]. HCP infers a set of artifactual covariates, by modeling such covariates using a prior that depends on linear combinations of known covariates. In a sense, by using the known covariates as training examples, HCP attempts to learn variability *patterns* that are likely to be artifactual, instead of merely finding factors that can explain the most variability. Based on the strength of the prior, HCP can balance the tradeoff between removing “narrow” and “broad” components that exhibit similar patterns to the covariates', and more major components that do not. Further, HCP's inferred covariates can also capture additive relationships between technical covariates that lead to spurious correlations (e.g., additive effects of gender and age).

Using the two RNA-seq datasets, we apply HCP and existing normalization methods to two tasks: (1) cis-eQTL detection, and (2) co-expression estimation. In accordance with previous studies with microarrays (e.g., [7]), our experiments confirm that all methods that remove hidden covariates greatly improve both the power of and consistency of cis-eQTL detection with RNA-seq data – with some small differences in performance. However, on a second task of constructing meaningful co-expression networks, blindly removing hidden components is not ideal – in this scenario, HCP outperforms simpler methods that make less stringent assumptions about the hidden covariates.

## Methods

In the following section, we will first introduce terminology for defining the expression normalization problem, and then present a unified residual framework for understanding the existing approaches. Then we present our new normalization method as derived from a natural extension to this framework.

### Unified Representation of Existing Normalization Methods

The goal of normalization is to decouple the true expression signal from known and unknown artifacts in the data. To do so, most existing methods (e.g., [6]–[8], [10], [11], [13], [15,]) assume that the observed expression measurements can be modeled in log-space (logarithm of read counts) using a Gaussian distribution with an expectation that depends linearly on the known and hidden covariates. Using this framework, the “true” expression measurements can be obtained by subtracting off the effects of such known and hidden covariates. Specifically, an expression dataset over 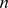 subjects and 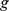 genes (or transcripts), as summarized in the 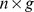 matrix 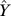, is modeled as follows:
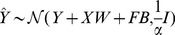
(1)where 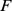 is a matrix of known covariates (also referred to as factors), 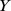 is the true, and unobserved, expression dataset, 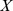 is a matrix of hidden covariates, and 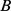 and 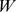 are matrices that model the effect of known and hidden covariates for each gene. In this setting 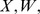 and 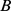 are unknown and must be estimated. Once the unknowns are estimated, they are removed from the data, and the true expression signal is computed as the residual 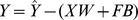. The primary differences between the existing methods are in (i) the types of covariates that are included in 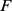, (ii) the assumed priors (or equivalently, the regularization form) on 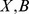 and 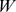, and (iii) the optimization algorithm used to fit the model parameters.

We note in the case of RNA-seq data, several authors have proposed to model the raw read counts using Poisson or negative binomial distributions (e.g., [16]); however, as described in the next section, we chose to model the logarithm of counts with a Gaussian distribution, as a well-studied downstream analyses that we consider is performed on ranks and not the actual number of reads (e.g., using Spearman correlation to identify eQTLs, see Methods). Additionally, Gaussian likelihood is widely used by practitioners (after suitable data transformation, e.g., [2], [17]–[19]) as it results in analytic solutions for the model parameters, considerably reducing the computational complexity. Similar analysis and insights could be applied to other distributional choices for the likelihood.

As further detailed in the Extended Methods, most existing methods can be derived from one of three special cases of the above formulation: (i) assuming only known covariates, (ii) assuming only unknown covariates, and (iii) assuming both known and unknown covariates. In each case, once the relevant unknown parameters are estimated, the corresponding artifactual effects are subtracted from the data.

For example, in the simplest scenario where we assume no hidden covariates, as in the ComBat method [15] or the regression method in [20], we only need to estimate the matrix 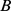, which models the effect of known covariates on the expression of each gene. Known covariates can include technical factors such as batch or sequencing lane, or biological factors such as age, or gender. The main limitation of the known-covariates-only methods is by relying solely on accurate measurements of known covariates, they cannot remove artifactual patterns that are not measured. In the second scenario, where we only assume the presence of hidden covariates, as in SVA [6], RUV [11], or sparse factor analysis [21], we need to estimate hidden covariates (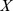) and their effects on each gene (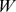). Depending on the priors placed on these parameters, we can derive various versions of the matrix factorization problem, including singular value decomposition (SVD), and factor analysis. The goal of matrix factorization in general is to find a low-dimensional representation of a high-dimensional dataset. In this way, the inferred hidden covariates summarize broad patterns that effect a large number of genes in the same way. Other related approaches include various Linear Mixed Models (LMMs), such as EMMAX [9] or PANAMA [13], that model the effect of hidden covariates implicitly by instead representing the broad correlation structure across individuals (see Extended Methods).

Finally, when assuming both known and hidden covariates (e.g. [7]) we need to simultaneously estimate hidden covariates (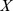), their effect on each gene (summarized by columns of 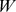), and the effect of known covariates on each gene (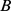). In this setting, including the known covariates allows the model to also remove narrower artifactual patterns (in addition to the broad patterns captured by the hidden covariates), as long as such patterns are exactly captured by the known covariates and result in variability in the expression measurements. For example, PEER [7] is a special case of this scenario. An additional assumption of PEER is that each gene is affected by a limited (“sparse”) set of hidden covariates. This assumption is represented by a particular form of prior, known as the ARD prior, on the matrix 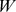.

In summary, it is important to note that methods that do attempt to infer hidden covariates (scenarios (ii) and (iii)) do so by assuming that such covariates represent broad patterns that affect many genes in the same way. This assumption leads to a matrix factorization problem where the goal is to find a low-dimensional representation of a high-dimensional dataset (either the original data, or the residual after correcting for known covariates). Effectively controlling for the complexity of such models then becomes the main determinant of their performance, since such models, in theory, are flexible enough to explain all the variability in the data by means of the hidden covariates alone. In the simplest form of matrix factorization such as SVD, model complexity is controlled by just one parameter 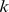 that determines the number of hidden covariates. Other models can have additional parameters that also penalize the norms of each hidden covariate or their effects, and in such models, complexity is not only determined by 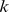 but by the simultaneous setting of all the parameters.

### Hidden Covariates with Prior (HCP)

The statistical model behind HCP is motivated by two observations. First, in expression data in general, known artifacts are often correlated with multiple non-consecutive principal components (PCs), reflecting the fact that artifacts can have both broad and narrow effects on expression; this observation has motivated post-hoc methods that preferentially remove PCs that are to some extent correlated with known confounders. Second, a possible scenario that the existing methods do not address is partial (or “noisy”) prior knowledge about possible confounding effects. For example, assume that we would like to account for smoking, and remove its effect, but our prior knowledge about the subjects' smoking behavior is incomplete because of reporting bias (some subjects do not report their smoking activity). Similar motivations have recently also inspired a method [22] for inferring the activity of cellular processes in individuals while using possibly noisy knowledge about pathway membership for genes.

Specifically, HCP infers a set of of artifactual covariates, by modeling such covariates using a prior that depends on linear combinations of known covariates: 
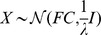
, where 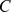 is an estimated matrix with 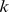 columns. By adjusting the strength of this prior, HCP's objective function allows us to represent the tradeoff between removing major trends in the expression patterns, and preferring the removal of known covariates. Importantly, HCP can also learn *patterns* of variation that are likely to be artifactual (either broad or narrow), based on the types of variability patterns that are captured by the known covariates. Using this prior, HCP will preferentially remove components with similar patterns to the covariates, allowing us to remove narrower components that are known to be artifactual, and more major components that are not (within a parameterized tradeoff scheme).

An alternative to the above approach would be to only assume that each hidden covariate has a prior Gaussian distribution with an expectation that depends on a known covariate (as opposed to linear combinations of known covariates), which gives rise to the expression 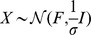. However, HCP has two main advantages over such a model: (i) HCP can summarize and collapse a possible set of redundant covariates to a smaller set of relevant covariates, and (ii) HCP can detect patterns that are associated with linear combinations of known covariates (e.g., additive effects of age and gender), that could also result in spurious correlations in the data.

HCP accepts a set of known covariates in order to estimate hidden covariates. Such known covariates can be any measurable factors that can introduce variability in expression measurements (for example, sequencing depth). As with the PEER method [7], it is also possible to use HCP without any known covariates: in such a scenario, both methods become equivalent to a matrix factorization method that infers principle components with broad effects on expression measurements. An additional advantage of HCP is its low computational cost: though the resulting objective is not jointly convex in 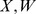, and 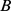, it is convex in each alone given a fixed setting of the rest. Therefore, this objective can be efficiently optimized by using a simple coordinate descent algorithm. In all of our experiments, computing the HCP solution required less than one minute of computational time on a standard desktop computer.

## Results

### RNA-seq Datasets

In our experiments we use two RNA-seq datasets; we will refer to these as Pickrell data [2] and Montgomery data [14].

The Pickrell RNA-seq data consists of data for 69 Yoruba HapMap cell lines (LCLs), and is available from http://eqtl.uchicago.edu/RNA_Seq_data/. We obtained the raw number of reads per exon, and aggregated the reads for each gene by taking the union of all reads mapped to its exons (for details of the read mapping procedure see [2]). All samples were done in replicates; in our analysis, we consider the replicate that has the largest number of mapped reads. We only considered genes with at least 30 reads in 10 individuals, which resulted in 12,569 expressed genes. The genotype data for 54 of these individuals is available from the pilot 1000 Genome project: ftp://ftp.1000genomes.ebi/ac.uk/vol1/ftp/pilot_data.

The Montgomery RNA-seq data consists of 60 Caucasian (i.e., the CEU population) HapMap cell lines (LCLs), and is available from http://jungle.unige.ch/rnaseq_CEU60/. Mapping and read quantification per exon was done as in [23]. As above, we obtained the raw number of reads per exon, and aggregated the reads for each gene by taking the union of all reads mapped to its exons. We only considered genes with at least 30 reads in 10 individuals, which resulted in 14,573 expressed genes. The genotype data for 58 of these individuals is available from the pilot 1000 Genome project: ftp://ftp.1000genomes.ebi/ac.uk/vol1/ftp/pilot_data.

**Figure 1:**
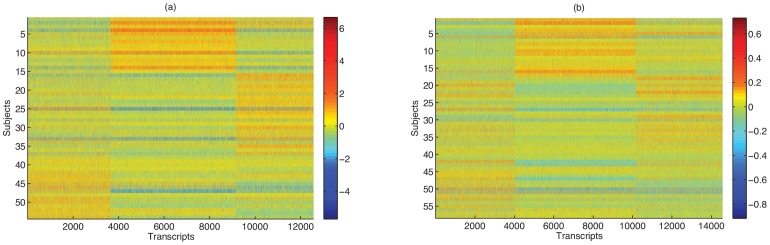
Heatmap of RNA-seq data from (a) Pickrell data and (b) Montgomery data. Rows represent subjects (or individuals) and columns represent genes. Using k-means clustering on corresponding RPKM normalized data, subjects are grouped in three clusters and genes are also grouped into three clusters. As observed later, these broad clustering patterns are primarily driven by confounding factors such as sequencing depth.

For visualization purposes, we applied k-means clustering to each dataset, using standardized, logarithm-transformed RPKM values, where each gene has zero mean and constant variance, to cluster subjects and genes. As shown in Figure 1, we observe strong trends in the data, represented by broad clustering patterns in both datasets. These clusters are present in both raw and RPKM data. As it turns out, SD is the main covariate that significantly correlates with this broad pattern (e.g., 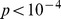 with the first, fourth, and twelfth PCs in the Pickrell data). Obviously, removing such artifactual trends will reduce, or remove, spurious correlations, and is thus a key aspect of any analysis that involves generalizing over genes or subjects. We note that from Figure 1, though, it is evident that simple scaling normalization methods such as RPKM or TMM [24], or rank-based normalization (e.g., Quantile normalization), cannot remove such broad clusters, as these methods preserve the ranking of the genes in each subject. One major explanation for this is that SD appears to affect different genes differently, which cannot be accounted for by such methods.

### Known Technical Covariates

As we describe in the next section, we applied HCP and several other methods that can be derived from the unified framework in Equation (1) to normalize Pickrell and Montgomery RNA-seq datasets. For methods that can use known covariates (like HCP, PEER [7], and a simple ridge regression method), we identified three technical covariates that can introduce subject-specific variability: (i) total number of reads (i.e., SD), (ii) subject-specific GC bias, and (iii) subject-specific gene-length bias. We describe each of these covariates below. (These three covariates are over subjects (or individuals), and constitute the columns of the matrix 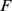 with dimensionality 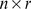 where 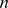 is the number of individuals and 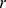 is the number of known covariates, which is 3 in our case).

We note that the models we consider correct systematic biases that vary over subjects (i.e., subject-specific covariates), and do not consider covariates that only vary over genes and are constant for the same gene in different subjects (e.g., direct effect of gene length on reads mapped to each gene which is considered in RPKM correction [25]). This is because we are not interested in the absolute expression level of each gene, and our analyses consist of identifying biological variability over *subjects*. Therefore, any bias that affects all subjects by the same factor (e.g., constant gene length for a given gene which affect the number of mapped reads) will not impact the results, and will simply be subtracted out when standardizing each gene.

We construct SD as the total number of mapped reads to exons per-subject. We obtain subject-specific GC as the total variation in read counts (in log space), per-subject, that can be explained by GC content. To do so, we compute the correlation coefficient between 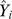 and 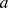 for each subject 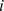, where 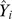 is a vector with elements representing the logarithm read count per gene, and 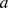 is a vector with elements representing the percent GC content per gene (i.e., 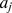 is percent GC content of gene 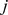). We considered subject-specific GC bias as a covariate as it was previously shown that GC bias can vary based on sequencing lane and so can be thought of as a subject-specific covariate [2]. We also suspected that a subject-specific gene-length covariate may capture technical differences in terms of the initial RNA quality. Similarly to the subject-specific GC bias, we obtain subject-specific length bias, for subject 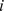, by correlating 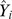 with 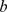, where 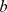 is a vector representing the length (in bases) for each of the genes.

The specific choice of the three known covariates was due to limited knowledge about other measured covariates in these two datasets, and that previous publications have shown that these types of covariates result in expression variability in RNA-seq data (in particular sequencing depth, and per-subject GC bias). There are many other technical or biological covariates, such as RIN, batch number, time of day, BMI and so on, that can affect expression, and should be utilized if available. Additionally, one can also include non-linear functions of the available covariates so as to be able to detect non-linear effects of the confounding factors.

## Evaluating Normalization Methods

In the following section, we compare various normalization methods in two different tasks: (i) identifying cis-eQTLs (ii) and constructing accurate co-expression networks.

### Identifying cis-eQTLs Using RNA-seq Data

We evaluated the residuals resulting from each normalization method by computing the number of significant associations between genes and SNPs that lie within 100Kb from the gene's transcription start site (TSS) (i.e., cis-eQTL analysis). SNPs that are within 100Kb of multiple genes as assigned to the closest gene. This type of evaluation based on cis-eQTL discovery is used in many previous eQTL studies [7], [8], [12]; unlike trans-eQTLs (association between SNP and distal genes), an increase in the overall number of significant cis-eQTLs is unlikely to arise from systematic artifacts because the particular genetic variation that impacts each gene is specific to the gene (i.e., a “narrow” component), and further, normalization procedures *remove* variable components of the data (and do not add variability), and therefore it is very unlikely to falsely introduce spurious associations. Following standard procedure [12], [14], we computed a p-value for the Spearman correlation coefficient for each gene and each nearby (cis) SNP. We accounted for multiple hypothesis testing using false discovery rate (FDR) [26] (applied to all tested SNP-gene pairs), and report the number of discoveries at 1%, 5%, and 10% FDR.

**Figure 2:**
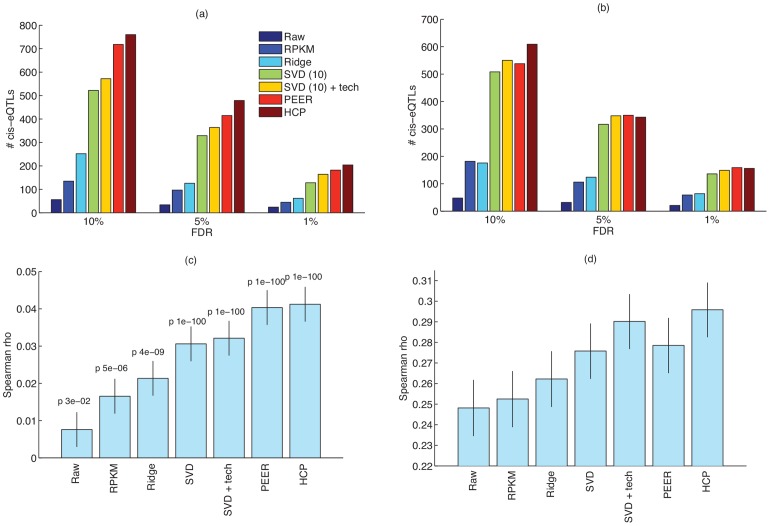
Detection of cis-eQTL on (a) Pickrell and data (b) Montgomery data. Correlation between (c) SNP-level p-values and (d) gene-level p-values in Montgomery and Pickrell datasets. P-values for the correlation coefficients for SNP-level comparisons are show on top of each bar. For the gene-level comparison, all p-values for the correlation coefficients are smaller than 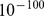. Error bars show the 90% confidence intervals for the correlation coefficients.

Figure 2 shows the number of cis-eQTLs that were detected using (i) the raw data, (ii) RPKM data, (iii) ridge regression (as in Equation 2), (iv) SVD, (v) ridge regression to remove known covariates followed by SVD (SVD + tech) (a simplified approximation to PEER) (vi) PEER, and (vii) our HCP method. We set the model parameters on one dataset (Montgomery), and use the same setting on the other dataset – we obtained similar parameter settings when using Pickrell data as the “training data” for choosing the parameter settings. For SVD, we investigated several settings for 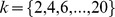 (the number of components that are removed), and observed the best performance for 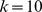 (shown in Figure 2). PEER also requires setting 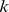, however, we noticed negligible difference for settings of 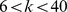 and so selected 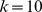 for both datasets. For our HCP method, we set 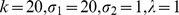, based on cross-validation on Montgomery data, and use the same settings of the parameters for all experiments.

As shown in Figures 2a and 2b, HCP improves slightly on PEER, and both methods increase the number of detected cis-eQTL by three to four folds compared to the RPKM data. Although the improvement of HCP over PEER is small, HCP is much faster: whereas PEER required at least 20 minutes of computation time (we used the C implementation of PEER provided by the authors, using the default thresholds on convergence), we can solve for the HCP solution in less than a minute on a standard desktop computer. This figure also illustrates that simpler methods, like ridge regression, that do not attempt to remove hidden covariates perform much worse than the other methods. In addition to ridge regression, we also investigated the performance of other “known covariates only” approaches that use different priors on 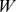. In particular, an element-wise sparsity prior, using the 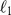 norm to regularize 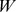, or low dimensionality prior, using the nuclear norm to regularize 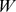, did not improve the performance of the “known covariates only” approach (data not shown). As demonstrated by Figures 2a and 2b, joint estimation of known and hidden covariates (as in PEER and HCP) tends to result in better performance compared to simply removing the top principal components of the data.

Using the HCP model, we find that 74% of the variance of removed expression (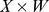) can be explained by known factors, where 35–75% of variance of each hidden factor can be explained by a combination of known technical factors, and an additional 5–20% of some factors can be explained by non-linear combination of these factors (based on five fold cross-validation). The remaining variance is likely to be due to other technical or biological factors that are not directly measured.

In addition to the eQTL test, we also compared the consistency of the eQTL discovery in the two datasets. To do so, we computed the Spearman correlation coefficient between the vectors of SNP (and also gene) p-values in the two datasets. There are 75,358 shared SNPs that are associated with a gene (i.e., cis-SNP), corresponding to 7,751 genes. As shown in Figures 2c and 2d, HCP along with PEER and SVD greatly improve the consistency of discoveries between these two studies.

#### Differences in cis-eQTL Discovery Based on Normalization

Having shown that accounting for both known and hidden factors greatly improves cis-eQTL discovery, two related questions naturally follow: (1) to what extent do the cis-eQTLs discovered by various methods overlap, and (2) to what extent are newly discovered cis-eQTLs detectable in the original, unnormalized dataset? To answer these questions, we first investigated the concordance between the list of cis-eQTLs produced by the different normalization methods. As a first analysis, we assessed the fraction of shared cis-eQTLs at 10% FDR between all pairs of normalized datasets. As shown in Table 1, all normalization methods re-discover a large fraction of cis-eQTLs that are discovered in the most basic version of the data (e.g., RPKM normalized). However, removing hidden covariates does not uniformly decrease the p-values and the discrepancy is not merely a thresholding issue. This is evident from from the low Spearman correlation coefficients between vectors of gene-SNP p-values obtained for RPKM and various versions of normalized data: the Spearman correlation coefficient between RPKM and SVD, PEER, and HCP, are 0.17, 0.29, and 0.3, respectively. Finally, though there is a large overlap between the discoveries made when using different normalization methods that estimate hidden covariates (e.g., Table 1: 73% of cis-eQTLs discovered by PEER are also discovered by HCP), normalization does result in differences in lists of prioritized cis-eQTLs (e.g., 30% of cis-eQTLs discovered by HCP are not discovered by PEER). So in short, the methods that account for hidden covariates do not produce completely different results compared to those that don't, and most of the discoveries made in baseline models (e.g., RPKM or Ridge) are also rediscovered by the more complex models. However, in additions to the baseline discoveries, hidden covariates methods discover statistically significant associations that were perhaps impacted by unknown confounding factors, which reduced the detection power.

**Table 1:** Fraction of shared cis-eQTLs at 10% FDR between pairs of various versions of normalized Pickrell RNA-seq data.

	RPKM	Raw	Ridge	SVD	SVD + tech	PEER	HCP
RPKM	1.00	0.83	0.78	0.83	0.84	0.91	0.86
Raw	0.35	1.00	0.78	0.78	0.77	0.90	0.90
Ridge	0.18	0.43	1.00	0.65	0.60	0.82	0.88
SVD	0.09	0.20	0.31	1.00	0.77	0.83	0.77
SVD + tech	0.08	0.18	0.25	0.70	1.00	0.74	0.70
PEER	0.07	0.16	0.28	0.60	0.58	1.00	0.73
HCP	0.06	0.16	0.29	0.53	0.53	0.70	1.00
							

Each row depicts the fraction of cis-eQTLs discovered using a particular normalization method that are also discovered in another normalized version. For example, the element at row *i* and column *j* (the 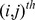 element) depicts the proportion of cis-eQTLs discovered using normalization method *i* that are also discovered in normalization method *j*.

### Accuracy in Co-expression Network Analysis

A correlation between two genes is often indicative of co-regulation, co-functionality, or a regulatory relationship. In fact, the “guilt-by-association” principle [27] is routinely applied to co-expression networks to identify genes with similar functionalities, or to predict function for unknown genes. However, technical artifacts in expression data that lead to spurious correlations between the subjects result in false positive associations between genes, reducing the accuracy of guilt-by-association based approaches.

As we showed in the last section, a normalization step that removes confounding variability in the RNA-seq data greatly improves the power of cis-eQTL detection. On the other hand, the correlation between genotype and expression are expected to be much narrower and weaker than correlations between expression levels of two co-functional or co-regulated genes, and it is not clear if an un-guided removal of the top principal components will also remove true biological variability that is not evident in the cis-eQTL detection task. In this section, we investigate the accuracy of co-expression networks constructed from Montgomery and Pickrell datasets in terms of reflecting co-functionality between genes, after the application of several normalization methods.

In particular, we investigate the consistency of the constructed co-expression networks with prior knowledge about co-functionality of genes, by attempting to use these networks as input to a gene function prediction algorithm. More specifically, we follow a standard approach (as in [28], also see [29]): we first construct a co-expression network from each dataset using the Pearson correlation coefficient. We then attempt to predict gene function from each network using a label propagation algorithm [30]. Specifically, from each network, we predict gene function for Gene Ontology (GO) [31] categories (downloaded September 2010) that have between 30–300 annotations. We excluded all annotations with an IEA evidence code, as these annotations are less reliable and not manually curated. We evaluated the performance using average area under the precision recall curve (AUP) in predicting each gene function in 5-fold cross-validation.

**Figure 3:**
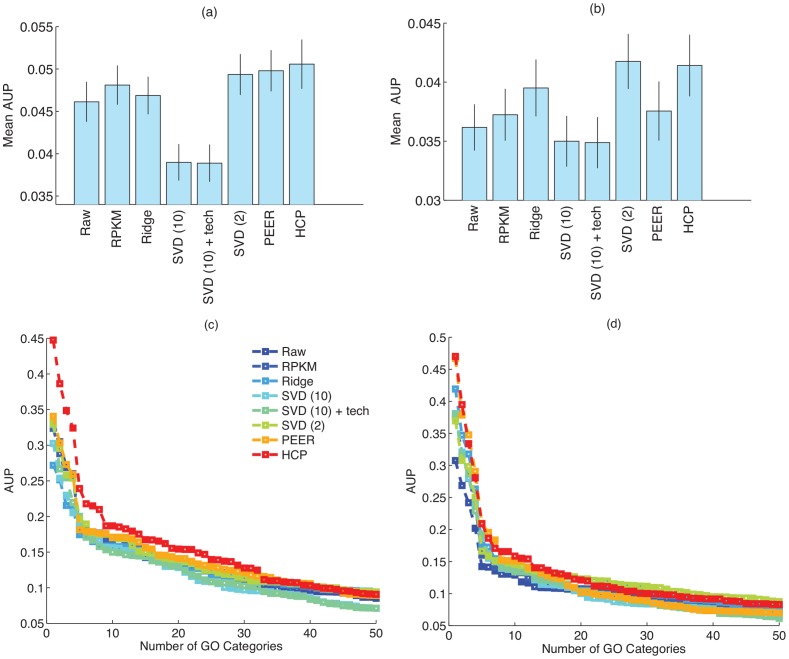
Mean average precision (AUP) in predicting gene function from co-expression networks constructed from various normalization methods, on (a) Pickrell, and (b) Montgomery data. The figure shows the performance of SVD at two different parameter settings (SVD (10) with 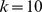, and SVD (2) with 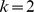). Error bars show the standard errors. Figure shows the cumulative performance for (c) Pickrell, (d) and Montgomery, datasets for the top 50 best predicted GO categories for each method.

**Figure 4:**
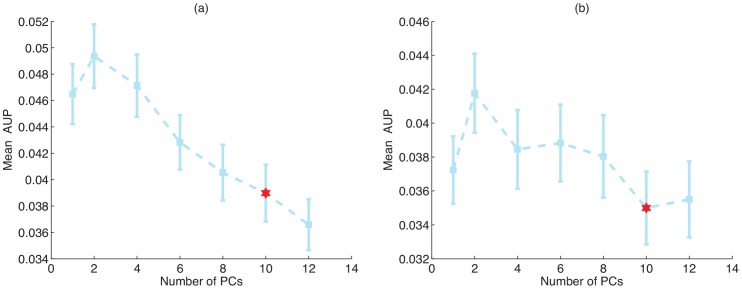
Performance of SVD on the GO prediction test with varying number of removed PCs (i.e., setting of 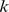) on (left) Pickrell and (right) Montgomery data. The red star marks the optimal setting of 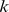 for the cis-eQTL task.

As a first analysis, for all the normalization methods we used the same parameter setting as in the previous section; by doing so, we also investigate the robustness of these methods with respect to the type of down-stream analysis performed on the normalized data. Figure 3 shows AUPs for each of the co-expression networks. Since the performance of SVD significantly varies based on the setting of its parameter 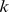 (shown in Figure 4), the final performance comparison in Figure 3 shows both the best setting of 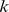 for cis-eQTL detection, and for the GO prediction test (SVD (2) and SVD (10), respectively). Unlike the cis-eQTL detection task, an unguided removal of top principal components, where 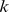 is maximized for the performance on cis-eQTL task, significantly reduces the accuracy of co-expression networks compared to the RPKM data. In contrast, PEER and HCP still remove hidden covariates using the same setting optimized for the cis-eQTL task, resulting in prediction of gene function. Therefore, compared to principal components, the hidden components that are removed by PEER and HCP are more likely to be artifacts, and these methods are less prone to overfitting.

## Discussion and Conclusion

RNA-sequencing is becoming widely adopted in large-scale transcriptomic studies, and specifically in genetics of gene expression studies (eQTL studies) (e.g., Geuvadis project http://www.geuvadis.org/web/geuvadis/RNAseq-project and GTEx project [32]). A major factor in deriving *meaningful* biological knowledge from transcriptomic studies that are based on healthy subjects or unstimulated data, where there is no apparent response component, is correcting for unwanted variability that stems from technical factors or unwanted biological variation. The impact of such factors on expression variability is often larger than those that derive the biological questions of interest, and if unaccounted for, results in spurious associations, or a loss in power. For instance, previous eQTLs studies have shown that, depending on the size of the study, removing even up to 50 PCs of expression data improves identification of genetic associations between cis-SNPs and expression variability of *local* genes [33].

Consistently with previous studies on microarrays, we find that when using RNA-seq data, removing broad expression trends (either PCs of expression data, HCP or PEER hidden factors) results in a drastic increase in the number of cis-eQTL discoveries (between 2 to 3 fold). Further, we find that the improvement in the number of cis-eQTL discoveries is not very sensitive to the exact number of PCs that are removed, so long as a sufficiently large number of PCs are removed (e.g., accounting for 60–80% variance in expression) – for example, here, we saw that removing between 8–16 PCs resulted in similar performance. For the cis-eQTL analysis, we inferred and removed 20 HCP factors. However, the same conclusion is unlikely to hold for identification of trans-eQTLs. In this study, we did not have sufficient power to identify trans-eQTLs, and as an alternative, we investigated the effect of normalization on accurate estimation of co-expression networks. Because *master regulators*, or *hub* genes, impact the expression levels of many target genes, their expression profiles are likely to be correlated with the expression PCs. This is also expected to hold for trans-eQTLs, because they are likely to impact the expression levels of many target genes. In our co-expression network analysis, we found that accurate estimation of co-expression between genes is much more sensitive to removing expression PCs; for instance removing 10 expression PCs (as done for cis-eQTL analysis) resulted in a drastic loss of accuracy in identifying known co-functional interactions (Figure 4). On the other hand, the parameter settings of HCP and PEER are more robust across the two types of downstream analyses that we investigated; removing the same number of hidden components improves performance in both analyses. This observation indicates that the hidden components removed by HCP or PEER more precisely capture the artifactual component, and are less likely to remove biological signal. However, from a technical perspective, there are several fundamental difference between PEER and HCP. First, HCP prefers to identify components that resemble known artifacts, either linearly or non-linearly. This distinction will be important when we have a *noisy* knowledge of the true artifacts. Second, HCP can potentially account for a large set of correlated known covariates, and summarize their impact on expression variability by a few HCP factors.

In summary, we have presented a unified residual normalization framework that encompasses most existing methods that attempt to account for known and/or hidden confounding covariates, and to remove their effects from expression data. As discussed, the framework enables a better understanding of the similarities and differences between the various existing approaches. Further, using this framework, we presented a novel normalization method called HCP. Whereas the existing approaches assume that one of the most distinguishing features of the hidden covariates is that they capture very broad patterns in the data, HCP attempts to learn the types of patterns (either broad or subtle) that can be artifactual. Based on the experiments conducted, we see that normalization models that account for both known and hidden covariates drastically reduce spurious correlations, and lead to more accurate co-expression networks and cis-eQTLs. Further, a model like that of HCP, that can account for both broad and subtle *patterns* of correlation in order to infer hidden covariates yields an effective and flexible method for correcting systematic biases.

## Extended Methods

In the next few sections we consider special cases of the unified normalization framework, summarized by Equation (1): (i) no hidden covariates, (ii) no known covariates, (iii) and both known and hidden covariates, and discuss the resulting normalization methods. As we will show, most existing normalization methods fall under one of these scenarios.

### Known Covariates

In the simplest case where we assume no hidden covariates, we can solve for 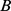 by minimizing the negative log likelihood of the data:
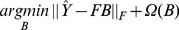
(2)where 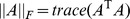 is the squared Frobenius norm of 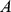, and 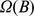 is the penalty placed on 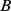. For example, placing a Gaussian prior on 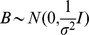 (also known as the ridge penalty), equivalent to 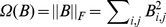, often alleviates over-fitting and results in better performance on test data. Here, we can analytically solve for 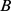 and obtain the residual 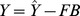.

The RPKM normalization method [25], a standard method for RNA-seq data, along with TMM [24], can be expressed as a solution to the above problem. In particular, RPKM normalization accounts for SD and gene length bias by dividing all reads assigned to a gene 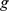 (or transcript) in subject 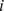 by (i) 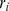, the SD of subject 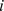 (in millions) and (ii) 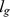, the length of gene 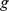 (in thousands of base pairs). Therefore, the logarithm of the RPKM value is given by 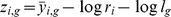, where 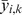 is the logarithm of number of reads mapped to gene 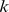 in subject 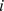. So we can derive the RPKM values from the residual model by assuming that sequencing depth has a constant effect (i.e., the same regression coefficient) on all genes. Unlike the RPKM normalization, the regression problem posed in Equation (2) allows the total number of reads and gene length to affect different genes differently, which will turn out to be important. Other related approaches that can be derived from Equation (2) include the linear regression framework proposed by [20], and the Empirical Bayes regression method in ComBat [15]. In the latter, the regularization term 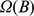 is designed so that multiple genes share the same parameter. This choice helps avoid over-fitting in small 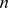 regime.

### Hidden Covariates

In the second scenario, where we only assume the presence of hidden covariates, depending on the constraints placed on 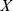 and 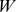, we can derive various versions of the matrix factorization problem, including singular value decomposition (SVD), factor analysis (FA), and non-negative matrix factorization (NNMF). For example, for SVD optimizes:
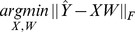
(3)

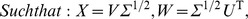


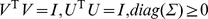



In this setting, the number of columns of 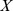, 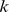, is unknown. Typically, 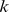 is set by the user to achieve a fixed level of “explained variability”. Alternatively, 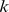 can also be tuned simultaneously as part of the same optimization problem (e.g., by introducing a group penalty on 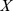 that penalizes the sum of norms of columns of 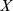 [13]), or can be set based on a significance test (e.g., permutation test in SVA [6], or significance of principal components in PCAsig [7]). Other related approaches to SVD include SVA [6] and RUV [11] which explicitly use prior knowledge to determine which principle components are artifactual. For example, SVA consists of a regression step first to remove the effects of interest from the observed expression dataset, and then it performs an SVD on the residual dataset – in this way, the effect of interest are not accidentally removed by the Principle Components. RUV uses control genes, which, to some extend, are expected to have constant expression across individuals, to identify variability patterns that are likely to be artifactual. In addition, minor alterations to the above allow us to derive several variations of the FA model. For example, FA assumes 
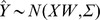
 with a diagonal matrix 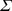 that is estimated along with 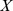 and 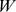, and sparse FA assumes an additional sparse penalty on 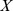 (e.g., element-wise 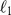 penalty on 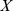) [21].

### Known and Hidden Covariates

Finally, assuming both known and hidden covariates, we need to estimate 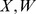 and 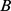:
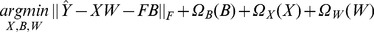
(4)where 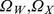 and 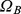 are the penalties on 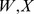 and 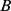, respectively. Assuming the same orthogonal constraints on 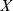 and 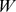 as in Equation (3), we can solve Equation (4) by iterating between (i) solving for 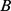 using the current estimate of 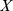 and 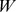, and (ii) solving for the SVD of 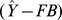 to set 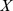 and 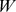. As another example, the PEER [7] method essentially solves the above objective, while assuming prior Gaussian distributions with zero-means on 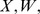 and 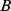, and a sparsity-type prior on 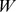.

We can also derive various forms of Linear Mixed Models (LMMs) such as EMMAX [34], and PANAMA [13] from the same framework. In particular, LMMs optimize a slightly different objective, by first specifying a Gaussian prior on 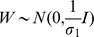 and 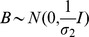 and then integrating out 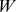 and 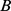 from the likelihood 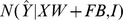 to obtain the final objective function. Recall that optimizing the likelihood 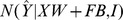 with respect to 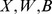 is equivalent to finding the solution to Equation (4). LMMs model the effect of 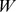, and 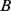, implicitly through the initial integration of the likelihood. Such approaches are typically used to detect eQTLs while correcting for population structure in genome wide association studies (GWAS), where columns of 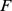 represent the first few principal components of the SNP data.

### Hidden Covariates with Prior

HCP can also be derived as a natural extension of this unified framework that is not fully encapsulated by the existing methods. In particular, optimizing the log likelihood of the data, as modeled by HCP, results in the following objective:
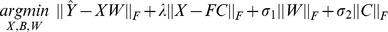
(5)where 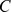 is the unknown matrix of effect of known factors 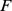 on unknown factors 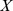, and 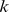, 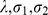 are the model parameters. Like any hidden factor model, increasing 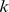 increases the variability in 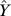 that can be explained by the model. However, in addition to the setting of 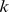, the model's capacity and performance depends on 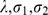. In our experiments, we set the model parameters using cross-validation based on a particular task of interest (e.g., set the parameters to maximize the number of recovered cis-eQTL on some portion of the genes, and use the same setting for all other genes); for comparison we use the same procedure for setting 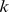 and other relevant parameters for the other models (e.g., SVD and PEER) that estimate hidden covariates. In all of our experiments, computing the HCP solution required less than one minute of computational time on a standard desktop computer.

### Availability

Software is available from authors by request.
